# Translation and validation of PubMed and Embase search filters for identification of systematic reviews, intervention studies, and observational studies in the field of first aid

**DOI:** 10.5195/jmla.2021.1219

**Published:** 2021-10-01

**Authors:** Bert Avau, Hans Van Remoortel, Emmy De Buck

**Affiliations:** 1 bert.avau@cebap.org, Centre for Evidence-Based Practice, Belgian Red Cross, Mechelen, Belgium; 2 hans.vanremoortel@cebap.org, Centre for Evidence-Based Practice, Belgian Red Cross, Mechelen, Belgium; 3 emmy.debuck@cebap.org, Centre for Evidence-Based Practice, Belgian Red Cross, Mechelen, Belgium; Department of Public Health and Primary Care, Faculty of Medicine, KU Leuven, Leuven, Belgium; Cochrane First Aid, Mechelen, Belgium

**Keywords:** search filters, guideline development, study design filters, first aid

## Abstract

**Objective:**

The aim of this project was to validate search filters for systematic reviews, intervention studies, and observational studies translated from Ovid MEDLINE and Embase syntax and used for searches in PubMed and Embase.com during the development of evidence summaries supporting first aid guidelines. We aimed to achieve a balance among recall, specificity, precision, and number needed to read (NNR).

**Methods:**

Reference gold standards were constructed per study type derived from existing evidence summaries. Search filter performance was assessed through retrospective searches and measurement of relative recall, specificity, precision, and NNR when using the translated search filters. Where necessary, search filters were optimized. Adapted filters were validated in separate validation gold standards.

**Results:**

Search filters for systematic reviews and observational studies reached recall of ≥85% in both PubMed and Embase. Corresponding specificities for systematic review filters were ≥96% in both databases, with a precision of 9.7% (NNR 10) in PubMed and 5.4% (NNR 19) in Embase. For observational study filters, specificity, precision, and NNR were 68%, 2%, and 51 in PubMed and 47%, 0.8%, and 123 in Embase, respectively. These filters were considered sufficiently effective. Search filters for intervention studies reached a recall of 85% and 83% in PubMed and Embase, respectively. Optimization led to recall of ≥95% with specificity, precision, and NNR of 49%, 1.3%, and 79 in PubMed and 56%, 0.74%, and 136 in Embase, respectively.

**Conclusions:**

We report validated filters to search for systematic reviews, observational studies, and intervention studies in guideline projects in PubMed and Embase.com.

## INTRODUCTION

The development of evidence-based practice guidelines is a time-consuming process [1]. Evidence-based recommendations require the availability of the best available scientific evidence, collected through systematic literature searches [2]. The Belgian Red Cross specializes in the development of evidence-based first aid guidelines, both for use in Belgium and for use by partner Red Cross National Societies in Southern countries [3–7]. Guideline development processes of the Belgian Red Cross are facilitated by its Centre for Evidence-Based Practice (CEBaP) according to strict methodology, which is detailed in an online available methodological charter [8]. During the 2019 development of an advanced first aid manual for Sub-Saharan Africa and the 2020 updates of the basic first aid manuals for Flanders, Belgium, and Sub-Saharan Africa, a total of 490 evidence summaries were developed to inform practical recommendations. In sixty-seven of these evidence summaries, methodological search filters for the identification of systematic reviews [9], intervention studies [10], and observational studies [9] in PubMed and Embase were used to decrease the number of records to screen. The search filters used were based on filters originally designed by the Scottish Intercollegiate Guidelines Network (SIGN) [9] or the Cochrane Effective Practice and Organisation of Care (EPOC) group [10] for use in Ovid MEDLINE and Ovid Embase but had not been validated for use in PubMed or Embase.com. These filters have been translated, with minor adaptations, and used in our organization in the absence of properly validated search filters specifically designed for use in PubMed and Embase.com [11].

Search filters are standardized combinations of search terms (indexing terms and/or free text words) to identify records with a specific feature, in this case aspects of study design [12]. Other examples of search filters include geographical filters [13] or topic search filters [14]. Ideally, search filters are validated, which means their performance in retrieving relevant records is tested against a “gold standard,” a known set of relevant records, thereby demonstrating that the filter reliably finds the evidence for which it is designed [15]. A methodological search filter can be seen as a “diagnostic test” to detect relevant records in a search [16]. An important measure of search filter performance is recall or sensitivity, which is the proportion of records from a gold standard that are retrieved when using a methodological search filter out of the total number of records in the gold standard [17]. In addition, we want to avoid screening as many irrelevant records as possible, which can be expressed by specificity, which is the proportion of irrelevant records that are not retrieved during the search with the filter out of the total number of irrelevant records in a search without the filter [18]. A different way of expressing the extent to which relevant records are retrieved and irrelevant records are excluded by a search filter is precision, which is the proportion of relevant records out of the total number of retrieved records [19], or the inverse of this, the number needed to read (NNR) to detect a relevant record [20].

There are typically two ways of composing a gold standard. One way is to hand-search a set of records (e.g., bibliographies of selected journals) [15]. The records that meet a certain criterion for relevance, such as study design, comprise the gold standard, and performance in retrieving these records through a database search using a search filter can be investigated. An alternative approach is to use the relative recall technique, where the gold standard is composed of studies meeting the relevance criteria in prior systematic searches where no search filter was used [21]. By running retrospective searches with and without the search filter, the performance of the search filter in retrieving gold standard records without retrieving too many irrelevant records can be assessed.

Given its recently obtained ISO 9001:2015 certification for the development of systematic reviews and evidence-based guidelines, CEBaP strives for a continuous quality improvement of its processes [22]. The current research is part of CEBaP's continuous quality improvement processes and aims to validate and, where necessary, also optimize (i.e., adapting the filters to increase performance) the study design search filters for systematic reviews, intervention studies, and observational studies in PubMed and Embase.com for future use in guideline development projects, thereby aiming to find a balance between recall and specificity.

## METHODS

### Description of the original search filters

The systematic review filters tested were translated from existing filters from SIGN, designed for Ovid MEDLINE and Ovid Embase, to PubMed and Embase.com syntax [9] (Table 1). In addition, minor adaptations were done to accommodate for indexing terms related to systematic reviews that were added to PubMed's MeSH tree and Embase's Emtree after the development of the SIGN filters. For PubMed, we included “Systematic Review”[PT] and “Systematic Reviews as Topic”[MeSH] in the filter. For Embase, we included ‘meta analysis (topic)’/exp, ‘systematic review (topic)’/exp, and ‘systematic review’/exp in the filter.

**Table 1:** Methodological search filters tested

Study design	PubMed	Embase
Systematic reviews	((“Meta-Analysis as Topic”[MeSH] OR meta analy*[TIAB] OR metaanaly*[TIAB] OR “Meta-Analysis”[PT] OR “Systematic Review”[PT] OR “Systematic Reviews as Topic”[MeSH] OR systematic review*[TIAB] OR systematic overview*[TIAB] OR “Review Literature as Topic”[MeSH]) OR (cochrane[TIAB] OR embase[TIAB] OR psychlit[TIAB] OR psyclit[TIAB] OR psychinfo[TIAB] OR psycinfo[TIAB] OR cinahl[TIAB] OR cinhal[TIAB] OR “science citation index”[TIAB] OR bids[TIAB] OR cancerlit[TIAB]) OR (reference list*[TIAB] OR bibliograph*[TIAB] OR hand-search*[TIAB] OR “relevant journals”[TIAB] OR manual search*[TIAB]) OR ((“selection criteria”[TIAB] OR “data extraction”[TIAB]) AND “Review”[PT])) NOT (“Comment”[PT] OR “Letter”[PT] OR “Editorial”[PT] OR (“Animals”[MeSH] NOT (“Animals”[MeSH] AND “Humans”[MeSH])))	((‘meta analysis (topic)’/exp OR ‘meta analysis’/exp OR (meta NEXT/1 analy*):ab,ti OR metaanaly*:ab,ti OR ‘systematic review (topic)’/exp OR ‘systematic review’/exp OR (systematic NEXT/1 review*):ab,ti OR (systematic NEXT/1 overview*):ab,ti) OR (cancerlit:ab,ti OR cochrane:ab,ti OR embase:ab,ti OR psychlit:ab,ti OR psyclit:ab,ti OR psychinfo:ab,ti OR psycinfo:ab,ti OR cinahl:ab,ti OR cinhal:ab,ti OR ‘science citation index’:ab,ti OR bids:ab,ti) OR ((reference NEXT/1 list*):ab,ti OR bibliograph*:ab,ti OR hand-search*:ab,ti OR (manual NEXT/1 search*):ab,ti OR ‘relevant journals’:ab,ti) OR ((‘data extraction’:ab,ti OR ‘selection criteria’:ab,ti) AND review/it)) NOT (letter/it OR editorial/it OR (‘animal’/exp NOT (‘animal’/exp AND ‘human’/exp)))
Intervention studies	*Original Filter:* ((“Clinical Trial”[PT] OR “Comparative Study”[PT] OR “Cross-Over Studies”[MeSH] OR “Clinical Trials as Topic”[MeSH] OR random*[TIAB] OR controll*[TIAB] OR “intervention study”[TIAB] OR “experimental study”[TIAB] OR “comparative study”[TIAB] OR trial[TIAB] OR evaluat*[TIAB] OR “before and after”[TIAB] OR “interrupted time series”[TIAB]) NOT (“Animals”[MeSH] NOT (Animals[MeSH] AND “Humans”[MeSH])))	*Original Filter:* (‘randomized controlled trial’/exp OR ‘clinical trial’/exp OR ‘comparative study’/exp OR random*:ab,ti OR control*:ab,ti OR ‘intervention study’:ab,ti OR ‘experimental study’:ab,ti OR ‘comparative study’:ab,ti OR trial:ab,ti OR evaluat*:ab,ti OR ‘before and after’:ab,ti OR ‘interrupted time series’:ab,ti) NOT (‘animal’/exp NOT ‘human’/exp)
	*Optimized filter:* ((“Clinical Trial”[PT] OR “Comparative Study”[PT] **OR “Evaluation study”[PT]** OR “Cross-Over Studies”[MeSH] OR “Clinical Trials as Topic”[MeSH] OR random*[TIAB] OR controll*[TIAB] OR “intervention study”[TIAB] OR “experimental study”[TIAB**] OR “comparative study”[TIAB]** OR trial[TIAB] **OR trials[TIAB]** OR evaluat*[TIAB] OR repeat*[TIAB] OR compar*[TIAB] OR versus[TIAB] OR “before and after”[TIAB] OR “interrupted time series”[TIAB]) NOT (“Animals”[MeSH] NOT (Animals[MeSH] AND “Humans”[MeSH])))	*Optimized filter:* (‘randomized controlled trial’/exp OR ‘clinical trial’/exp OR ‘comparative study’/exp **OR ‘controlled study’/de OR ‘evaluation study’/de OR ‘human experiment’/exp** OR random*:ab,ti OR control*:ab,ti OR ‘intervention study’:ab,ti OR ‘experimental study’:ab,ti **OR ‘comparative study’:ab,ti** OR trial:ab,ti OR trials:ab,ti OR compar*:ab,ti OR repeat*:ab,ti OR crossover:ab,ti OR ‘double blind’:ab,ti OR evaluat*:ab,ti OR ‘before and after’:ab,ti OR ‘interrupted time series’:ab,ti) NOT (‘animal’/exp NOT ‘human’/exp)
Observational studies	“Epidemiologic Studies”[MeSH] OR “case control”[TIAB] OR “case-control”[TIAB] OR ((case[TIAB] OR cases[TIAB]) AND (control[TIAB] OR controls[TIAB)) OR “cohort study”[TIAB] OR “cohort analysis”[TIAB] OR “follow up study”[TIAB] OR “follow-up study”[TIAB] OR “observational study”[TIAB] OR longitudinal[TIAB] OR retrospective[TIAB] OR “cross sectional”[TIAB] OR questionnaire[TIAB] OR questionnaires[TIAB] OR survey[TIAB]	‘clinical study’/exp OR ‘cohort analysis’/exp OR ‘case control’:ab,ti OR ‘case-control’:ab,ti OR ((case:ab,ti OR cases:ab,ti) AND (control:ab,ti OR controls:ab,ti)) OR ‘cohort study’:ab,ti OR ‘cohort analysis’:ab,ti OR ‘follow up study’:ab,ti OR ‘follow-up study’:ab,ti OR ‘observational study’:ab,ti OR longitudinal:ab,ti OR retrospective:ab,ti OR ‘cross sectional’:ab,ti OR questionnaire:ab,ti OR questionnaires:ab,ti OR survey:ab,ti OR ‘epidemiological study’:ab,ti

The intervention filters tested were based on an existing filter for intervention studies that was originally designed by the Cochrane EPOC group [10] for Ovid MEDLINE and Ovid Embase and was retrieved in 2009 but is no longer publicly available.

The observational filters tested were translated from existing filters from SIGN designed for Ovid MEDLINE and Ovid Embase to PubMed and Embase.com syntax [9].

### Validation of search filters

To test the performance of the methodological search filters, we used the relative recall technique [21]. This means we compared the retrieval of records included in existing evidence syntheses resulting from systematic searches without a methodological search filter, our reference gold standard, with the records retrieved when adding the translated search filters to these searches. To do so, the searches were rerun in a so-called “retrospective” search (i.e., rerunning the searches until the initial search date), first without the methodological filter and then with the methodological search filter.

#### Reference gold standard composition

The reference gold standards for this study were composed of records retrieved through systematic searches performed during the development of evidence summaries for our first aid guidelines. All evidence summaries informing the reference gold standards used for this study are available from the online CEBaP Evidence Summary Database [8]. A separate gold standard was developed per study design filter tested. To be included in a gold standard, records were

1. Retrieved from searches developed for an evidence summary informing our 2019 advanced first aid manual for Sub-Saharan Africa or the 2020 updates of our basic first aid guidelines for Flanders, Belgium, or Sub-Saharan Africa in PubMed or Embase
2. Identified as a relevant systematic review, intervention study, or observational study as judged by the reviewer of the evidence summary according to predefined study selection criteria described in CEBaP's methodological charter [23] and described in Appendix 1
3. Originally retrieved without using a methodological search filter

#### Calculation of outcome measures

Recall was calculated as follows:


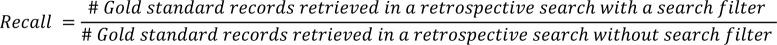



Specificity was calculated as follows:


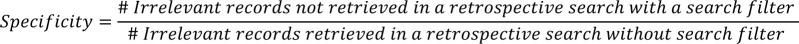



Precision, or positive predictive value, was calculated as follows:


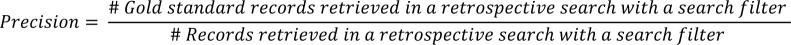



NNR was calculated as follows:


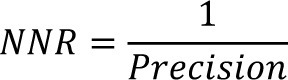



Records from different searches were accrued until a minimum of 70 relevant publications of a specific study design were included in a gold standard. This number differs from the 100 relevant records proposed by Sampson et al. to be included in a gold standard [21] but is sufficiently large to have an acceptable lower limit of confidence (>75%) when assuming 90% recall (i.e., we would have 95% confidence that at least 75% of relevant records will be retrieved in a given search) according to Flahault et al. [24]. Sampson et al. estimated that including 100 records would result in a lower confidence limit of 84% when assuming 90% recall but aimed to validate a search filter designed to maximize recall. This deviates from the purpose of our filter validation exercise, where we aim to balance recall and specificity.

Filters were considered sufficiently effective for use in literature searches for first aid guideline projects when recall was ≥85% (i.e., 85% or more relevant records are retrieved when searching with a methodological search filter) with a specificity of ≥45% (i.e., 45% or more of the irrelevant records are filtered out when searching with a methodological search filter).

### Optimization of search filters

In case our search filters did not reach the desired level of recall (≥85%) and specificity (≥45%) in both PubMed and Embase, we attempted to optimize them by looking for additional relevant search terms to be included in the filters. These were searched for in the indexing terms and title, abstract, and keywords of studies that were included in the reference gold standard but not retrieved when combining the original search with the search filter. Both the original search filters and newly optimized alternative search filters were then tested in an independent validation reference gold standard retrieved from a distinct set of first aid–related evidence summaries. The use of an independent validation gold standard is important, as this gives a more reliable estimate of how the filter performs in a real-world application and thus increases the generalizability of results [25].

## RESULTS

### Systematic review filter

#### PubMed

The reference gold standard consisted of 77 systematic review references, collected in 33 evidence summaries on different first aid topics. An overview of the evidence summaries can be found in Appendix 2. Of the 77 relevant systematic review references, 69 were retrieved when repeating searches with the PubMed search filter for systematic reviews, which resulted in a recall of 90%, specificity of 97%, and precision of 9.7% (Table 2). NNR to identify a relevant record decreased from 307 without the search filter to 10 with the search filter.

**Table 2:** Overview of systematic review search filter's performance in PubMed and Embase

	**PubMed**	
	Relevant records in retrospective searches (= gold standard, a+b): 77	Irrelevant records in retrospective searches (c+d): 23,201
Records retrieved with search filter (a+c): 713	True positives (a): 69	False positives (c): 644
Records not retrieved with search filter: 22,565	False negatives (b): 8	True negatives (d): 22,557
	**Embase**	
	Relevant records in retrospective searches (= gold standard, a+b): 70	Irrelevant records in retrospective searches (c+d): 30,670
Records retrieved with search filter (a+c): 1,192	True positives (a): 64	False positives (c): 1,128
Records not retrieved with search filter: 29,548	False negatives (b): 6	True negatives (d): 29,542

#### Embase

The reference gold standard consisted of 70 systematic review references, collected in 35 evidence summaries (Appendix 2). When using the search filter when repeating the searches in Embase, 64 out of 70 systematic review references were retrieved, resulting in a recall of 91%, specificity of 96%, and precision of 5.4% (Table 2). NNR decreased from 439 without the search filter to 19 with the search filter.

#### Filter performance consideration

The systematic review filter's performance was considered sufficient (recall ≥85% and specificity ≥45% in both PubMed and Embase) for use in first aid guideline projects. Therefore, the filter was considered validated, and no further optimization was pursued.

### Intervention study filter—derivation reference gold standard

#### PubMed

The reference gold standard consisted of 116 intervention study references, collected from 21 evidence summaries (Appendix 2). Using the search filter when performing retrospective searches led to identification of 98 out of 116 records, resulting in a recall of 85%, specificity of 68%, and precision of 3.1% (Table 3). NNR decreased from 85 without the search filter to 33 with the search filter.

**Table 3:** Overview of the original intervention study search filter's performance in PubMed and Embase in the derivation reference gold standard

	**PubMed**	
	Relevant records in a retrospective search (= gold standard, a+b): 116	Irrelevant records in a retrospective search (c+d): 9,717
Records retrieved with search filter (a+c): 3,218	True positives (a): 98	False positives (c): 3,120
Records not retrieved with search filter: 6,615	False negatives (b): 18	True negatives (d): 6,597
	**Embase**	
	Relevant records in a retrospective search (= gold standard, a+b): 103	Irrelevant records in a retrospective search (c+d): 14,484
Records retrieved with search filter (a+c): 5,437	True positives (a): 85	False positives (c): 5,352
Records not retrieved with search filter: 9,150	False negatives (b): 18	True negatives (d): 9,132

#### Embase

The reference gold standard consisted of 103 intervention study references, collected from 21 evidence summaries (Appendix 2). Addition of the search filter to the retrospective searches led to identification of 85 out of 103 records. Recall was 83%, specificity was 63%, and precision was 1.6% (Table 3). NNR decreased from 142 without the search filter to 64 with the search filter.

#### Filter performance consideration

The performance of the intervention search filter was considered insufficient. Further optimization by analyzing indexing terms, titles, abstracts, and keywords of missed reference gold standard records led to two optimized search filters (Appendix 3, Table 1), which were tested alongside the original search filters in an independent validation reference gold standard.

### Intervention filter—validation reference gold standard

#### PubMed

In the independent validation reference gold standard, consisting of 73 intervention study references from 18 evidence summaries (Appendix 4), the original search filter identified 66 out of 73 records. Recall was 90%, specificity was 68%, and precision was 1.7% (Table 4). NNR decreased from 145 without the search filter to 60 with the search filter.

**Table 4:** Overview of the original intervention study search filter's performance in PubMed and Embase in the validation reference gold standard

	**PubMed**	
	Relevant records in a retrospective search (= gold standard, a+b): 73	Irrelevant records in a retrospective search (c+d): 10,512
Records retrieved with search filter (a+c): 3,957	True positives (a): 66	False positives (c): 3,891
Records not retrieved with search filter: 6,628	False negatives (b): 7	True negatives (d): 6,621
	**Embase**	
	Relevant records in a retrospective search (= gold standard, a+b): 70	Irrelevant records in a retrospective search: 16,586
Records retrieved with search filter (a+c): 7,403	True positives (a): 63	False positives (c): 7,340
Records not retrieved with search filter: 9,253	False negatives (b): 7	True negatives (d): 9,246

The adapted filter performed better with respect to recall and identified 69 out of 73 records, with recall of 95%, specificity of 49%, and precision of 1.3% (Table 5). NNR using the adapted filter decreased to 79.

**Table 5:** Overview of the adapted intervention study search filter's performance in PubMed and Embase in the validation reference gold standard

	**PubMed**	
	Relevant records in a retrospective search (= gold standard, a+b): 73	Irrelevant records in a retrospective search (c+d): 10,512
Records retrieved with search filter (a+c): 5,444	True positives (a): 69	False positives (c): 5,375
Records not retrieved with search filter: 5,141	False negatives (b): 4	True negatives (d): 5,137
	**Embase**	
	Relevant records in a retrospective search (= gold standard, a+b): 70	Irrelevant records in a retrospective search (c+d): 16,586
Records retrieved with search filter (a+c): 9,217	True positives (a): 68	False positives (c): 7,371
Records not retrieved with search filter: 7,439	False negatives (b): 2	True negatives (d): 9,215

#### Embase

The independent validation reference gold standard consisted of 70 intervention study references, identified from 18 evidence summaries (Appendix 4). The original search filter identified 63 out of 70 records, which resulted in a recall of 90%, specificity of 56%, and precision of 0.85% (Table 4). NNR decreased from 238 without the search filter to 118 with the search filter.

The adapted intervention study search filter, on the other hand, identified 68 out of 70 records and had a recall of 97%, specificity of 56%, and precision of 0.91% (Table 5). NNR when using the adapted filter decreased to 136.

#### Filter performance consideration

Although NNR increased when using the adapted filter and the recall of the original filters was already high in the validation samples, the increased yield in gold standard records in our view justifies the altogether modest increase in records to screen.

### Observational filter

#### PubMed

The reference gold standard consisted of 83 observational study references, collected in 21 evidence summaries (Appendix 2). When using the observational study search filter, 71 out of 84 studies were retrieved, resulting in a recall of 85%, specificity of 68%, and precision of 2% (Table 6). NNR decreased from 132 without the search filter to 51 with the search filter.

**Table 6:** Overview of the observational study search filter's performance in PubMed and Embase

	**PubMed**	
	Relevant records in a retrospective search (= gold standard, a+b): 84	Irrelevant records in a retrospective search (c+d): 10,983
Records retrieved with search filter (a+c): 3,604	True positives a): 71	False positives (c): 3,533
Records not retrieved with search filter: 7,463	False negatives (b): 13	True negatives (d): 7,450
	**Embase**	
	Relevant records in a retrospective search (= gold standard, a+b): 77	Irrelevant records in a retrospective search (c+d): 17,019
Records retrieved with search filter (a+c): 9,078	True positives (a): 74	False positives (c): 9,004
Records not retrieved with search filter: 8,018	False negatives (b): 3	True negatives (d): 8,015

#### Embase

For Embase, the reference gold standard contained 77 observational study references, originating from 22 evidence summaries (Appendix 2). Addition of the observational study search filter resulted in 74 out of 77 studies retrieved, with a recall of 96%, specificity of 47%, and precision of 0.8% (Table 6). NNR decreased from 222 without the search filter to 123 with the search filter.

#### Filter performance consideration

The observational study search filter was considered sufficiently effective for use in first aid guideline evidence summaries. No further optimization was attempted.

## DISCUSSION

The production of 490 evidence summaries for a total of three first aid guidelines took approximately 3,045 working hours, of which approximately 822 hours were dedicated specifically to screening records retrieved with systematic literature searches at the title and abstract level. The current project aimed to increase time-efficient screening of potentially relevant papers in future first aid guideline projects by optimizing (where necessary) and validating methodological search filters that were used on an ad hoc basis in prior guideline projects. Search filters for systematic reviews and observational studies were considered sufficiently effective, whereas those for intervention studies were optimized to satisfactory levels for guideline project purposes. Theoretically, using these search filters could decrease the total screening time by approximately 42% if all searches were performed using a search filter. In practice, this yield will be less, as we aim for search filters to be used judiciously (i.e., only when appropriate) and depending on the yield of the search without the use of the filters.

### Study limitations

The current study has limitations. First, the filters evaluated in this paper were specifically tested on searches of evidence summaries used for first aid guideline projects. First aid is a thematically broad area of health [26, 27]. Diverse health problems are tackled in first aid guidelines, which all share an acute nature and laypeople as providers of care as common features. This means that our validation is fairly broad. Nevertheless, it remains unclear how the filters would perform in other areas of health. The systematic review and intervention study filter are specifically designed to detect human studies, which means they are not suitable to detect systematic reviews or intervention studies on animals. For this aim, other resources exist [28]. Second, we used the relative recall technique [21], which is a more “real-world” application of the use of search filters than composing a reference gold standard by hand-searching journals. This implies that only studies with thematic relevance are included in the gold standard, which leads to a lower precision and a higher NNR compared to a hand-searching gold standard due to the classification of studies with a correct study design but no thematic relevance as “false positives.” Third, systematic searches for guideline projects by CEBaP are more pragmatic than searches for systematic reviews in that they attempt to balance methodological rigor with the time constraints associated with guideline production [8]. Therefore, searches may be more focused when NNR is large and search results are screened by a single reviewer and checked by a second reviewer instead of independent screening by two reviewers. Fourth, the focused searches, and more importantly the fact that first aid is a notoriously understudied field, result in the fact that evidence summaries are often not abundant in evidence. However, by combining included studies from multiple evidence summaries, we were able to achieve gold standards with satisfactory sample sizes, though less than the 100 records suggested by Sampson et al. [21].

### Relation to published research on search filters

When comparing our results to those of others, it is clear that accurate identification of systematic reviews is of interest to many. Nevertheless, we are, to our knowledge, the first to validate search filters for systematic reviews for use in PubMed and Embase.com. Lee et al. compared several systematic review search filters, including the original Ovid MEDLINE and Ovid Embase version of the SIGN filter slightly adapted and tested by us [29]. Our findings in PubMed are comparable with theirs (i.e., recall of 87% in Lee et al. compared to 90% in our sample, with a specificity of 99% and 97%, respectively). However, the filter performs better in Embase in our sample compared to the results reported by Lee et al. in Ovid Embase (i.e., recall of 81% in Lee et al. compared to 91% in our sample, with a specificity of 99% and 96%, respectively). This may be due to different samples but may also reflect interface-specific differences in the syntax [30]. The SIGN filter was among the best regarding specificity but was outperformed in recall by others [31–33]. The balance of recall and specificity of this filter is considered sufficient for guideline projects.

Several authors have published validated methodological search filters for identification of randomized controlled trials, such as the Cochrane Highly Sensitive Search Strategy [19, 30, 34]. A recently published study by Waffenschmidt et al. designed search filters for the identification of controlled nonrandomized studies, comprising both nonrandomized intervention studies and controlled observational studies in MEDLINE [35]. To our knowledge, the intervention filter tested by us is the only one designed to identify intervention studies including, but not limited to, randomized controlled trials. The minor adaptations by us improved recall without compromising specificity too much.

Search filters for observational studies are much less studied than filters for systematic reviews or randomized controlled trials. The ISSG Search Filter Resource mentions several observational study filters for use in Ovid MEDLINE and Ovid Embase, but generally with untested performance [11]. A recent Cochrane methodology review on the topic identified two studies [36]. In one, Fraser et al. report high recall for search filters to detect observational studies of surgical interventions, but as these filters contained topic-specific terms, they are not practical in other thematic areas [37]. In another, Furlan et al. describe recall ranging from 48% to 93% using fixed search strategies for observational studies across four gold standards retrieved from the included references of four systematic reviews in Ovid PubMed and Ovid Embase without external validation in an independent reference gold standard [38]. The observational filters tested in this project for PubMed and Embase.com are among the first to be validated and demonstrate an acceptable performance for use in guideline projects.

Future research should aim to design and validate similar search filters for other scientific databases (e.g., CINAHL, ERIC, Web of Science), for which validated study design filters are scarce [11].

### Conclusion

To conclude, this work has validated methodological search filters for systematic reviews and observational studies and optimized methodological search filters for intervention studies in PubMed and Embase.com. The filters show an acceptable balance between recall and specificity for use in guideline projects, in particular in the field of first aid, but may be useful in other domains as well.

## Data Availability

Data associated with this article are available in the online appendix and at https://www.cebap.org/knowledge-dissemination/first-aid-evidence-summaries.

## SUPPLEMENTAL FILES

**Appendix 1:** Study design definitions & selection criteria used by CEBaP in first aid evidence summaries

**Appendix 2:** Search filter validation results

**Appendix 3:** Optimization of existing intervention study search filter for PubMed & Embase

**Appendix 4:** Validation reference gold standards composition
