# Supplementary material for: Translation and validation of PubMed and Embase search filters for identification of systematic reviews, intervention studies, and observational studies in the field of first aid
Source: J Med Libr Assoc. 2021 Oct 1;109(4):599–608. doi: 10.5195/jmla.2021.1219 (PMC8608173; doi:10.5195/jmla.2021.1219)
Supplement: Supplementary file 1 — Appendix 1: Study design definitions & selection criteria used by CEBaP in first aid evidence summaries [file jmla-109-4-599-s01.docx]

## Appendix 1: Study design definitions & selection criteria used by CEBaP in first aid evidence summaries

**Systematic review:** Inclusion of the studies of the systematic review if the search strategy and selection criteria are clearly described and if at least the Cochrane Library, MEDLINE and Embase are searched. Inclusion of a systematic review as a source of studies if the search strategy and selection criteria are clearly described and at least two databases have been searched, of which one is the Cochrane Library, MEDLINE, or Embase.

**Intervention study:** Inclusion in case of one of the following study types: (quasi or non-) randomised controlled trial (RCT), controlled before and after study, or controlled interrupted time series, and the data are available.

**Observational study:** Inclusion in case of one of the following study types: cohort and case-control study, controlled before and after study, or controlled interrupted time series, and the data are available.
