# Supplementary material for: Translation and validation of PubMed and Embase search filters for identification of systematic reviews, intervention studies, and observational studies in the field of first aid
Source: J Med Libr Assoc. 2021 Oct 1;109(4):599–608. doi: 10.5195/jmla.2021.1219 (PMC8608173; doi:10.5195/jmla.2021.1219)
Supplement: Supplementary file 2 — Appendix 2: Search filter validation results [file jmla-109-4-599-s02.docx]

## Appendix 2: Search filter validation results

### Systematic review search filter PubMed

| **Evidence summary [1]** | **Original search date** | **Number of records retrieved from PubMed without search filter** | **Relevant systematic reviews identified without search filter** | **Number needed to read without search filter** | **Number of records retrieved from PubMed when using filter** | **Relevant systematic reviews identified when using filter** | **Recall (%)** | **Number needed to read when using filter** |
| --- | --- | --- | --- | --- | --- | --- | --- | --- |
| Transport spinal injury | 20/11/2018 | 1812 | 9 | 32 | 58 | 7 | 78 | 8 |
| Psychological First Aid | 10/12/2018 | 129 | 4 | 553 | 5 | 3 | 75 | 2 |
| Amputated body part on ice | 4/01/2019 | 1105 | 2 | 201 | 15 | 2 | 100 | 8 |
| Burns: Chemical burn: irrigation | 22/11/2019 | 725 | 4 | 181 | 13 | 4 | 100 | 3 |
| Bites&Stings: Dog bite prevention | 27/09/2019 | 1131 | 2 | 566 | 10 | 2 | 100 | 5 |
| Bites&Stings: RF dog bite | 1/10/2019 | 444 | 1 | 444 | 6 | 0 | 0 | N/A |
| Bites&Stings: Pediculicides | 17/10/2019 | 818 | 2 | 409 | 14 | 2 | 100 | 7 |
| Bites&Stings: Washing/wet comb for lice | 17/10/2019 | 264 | 2 | 132 | 9 | 1 | 50 | 9 |
| Bites&Stings: Bednets for mosquitos | 30/08/2019 | 272 | 3 | 91 | 11 | 3 | 100 | 4 |
| Bites&Stings: Coils | 27/09/2019 | 280 | 1 | 280 | 8 | 0 | 0 | N/A |
| Bites&Stings: Environmental cleanliness & hygiene for scabies | 28/10/2019 | 683 | 2 | 342 | 12 | 2 | 100 | 6 |
| Bites&Stings: FA for snakebites | 4/10/2019 | 2640 | 1 | 2640 | 19 | 1 | 100 | 19 |
| Bites&Stings: Hot water for sting of marine animals | 18/07/2019 | 596 | 1 | 596 | 2 | 0 | 0 | N/A |
| Limbs: Muscle cramps massage | 10/03/2020 | 999 | 2 | 500 | 27 | 2 | 100 | 14 |
| Limbs: Stretching and warmup | 17/02/2020 | 1273 | 1 | 1273 | 83 | 1 | 100 | 83 |
| Limbs: Bracing and taping | 13/02/2020 | 267 | 4 | 67 | 22 | 4 | 100 | 6 |
| Limbs: Ice Elevation | 7/02/2020 | 1488 | 6 | 248 | 43 | 5 | 83 | 9 |
| Stomach and back: Dysmenorrhea | 25/05/2020 | 550 | 2 | 275 | 43 | 2 | 100 | 22 |
| Stomach and back: Lift technique | 26/05/2020 | 1049 | 4 | 262 | 42 | 3 | 75 | 14 |
| Stomach and back: Walking for back pain | 16/06/2020 | 668 | 1 | 668 | 29 | 1 | 100 | 29 |
| Stomach and back: Heat for back pain | 13/06/2020 | 1006 | 1 | 1006 | 43 | 1 | 100 | 43 |
| Poisoning: Safe storage of toxics | 2/04/2020 | 590 | 2 | 295 | 17 | 2 | 100 | 9 |
| Heat and cold: RF heatstroke | 15/05/2020 | 705 | 1 | 705 | 8 | 1 | 100 | 8 |
| Travel illnesses: Compression stockings | 22/08/2019 | 575 | 1 | 575 | 16 | 1 | 100 | 16 |
| Drowning prevention | 5/05/2020 | 336 | 2 | 168 | 15 | 2 | 100 | 8 |
| Telephone antenatal care | 17/08/2020 | 463 | 4 | 116 | 15 | 4 | 100 | 4 |
| Maternal sleeping position during pregancy | 3/08/2020 | 295 | 1 | 295 | 13 | 1 | 100 | 13 |
| Safe delivery kit | 6/08/2020 | 56 | 1 | 56 | 3 | 1 | 100 | 3 |
| Uterine massage | 6/08/2020 | 113 | 2 | 57 | 18 | 2 | 100 | 9 |
| Prevention of bullet wounds | 19/08/2020 | 767 | 2 | 384 | 23 | 2 | 100 | 12 |
| Burns: Aloe vera for burns | 31/08/2020 | 103 | 2 | 52 | 8 | 2 | 100 | 4 |
| Palpation for detection of fever | 7/9/2020 | 783 | 2 | 392 | 23 | 2 | 100 | 12 |
| Polymer-based ORS for diarrhoea | 15/9/2020 | 293 | 1 | 293 | 12 | 1 | 100 | 12 |

### Systematic review search filter Embase

| **Evidence summary [1]** | **Original search date** | **Number of records retrieved from Embase without search filter** | **Relevant systematic reviews identified without search filter** | **Number needed to read without search filter** | **Number of records retrieved from Embase when using filter** | **Relevant systematic reviews identified when using filter** | **Recall (%)** | **Number needed to read when using filter** |
| --- | --- | --- | --- | --- | --- | --- | --- | --- |
| Disinfection & wound irrigation of skin wounds | 10/01/2019 | 122 | 1 | 61 | 3 | 0 | 0 | N/A |
| Transport spinal injury | 20/11/2018 | 1717 | 4 | 343 | 55 | 4 | 80 | 8 |
| Psychological First Aid | 10/12/2018 | 131 | 4 | 33 | 5 | 3 | 75 | 2 |
| Amputated body part on ice | 4/01/2019 | 1277 | 1 | 1277 | 16 | 1 | 100 | 16 |
| Burns: Chemical burn: irrigation | 22/11/2019 | 388 | 2 | 194 | 9 | 2 | 100 | 5 |
| Bites&Stings: Dog bite prevention | 27/09/2019 | 1482 | 2 | 741 | 24 | 2 | 100 | 12 |
| Bites&Stings: RF dog bite | 1/10/2019 | 426 | 1 | 426 | 7 | 1 | 100 | 7 |
| Bites&Stings: Pediculicides | 17/10/2019 | 1336 | 2 | 668 | 25 | 2 | 100 | 13 |
| Bites&Stings: Washing/wet comb for lice | 17/10/2019 | 234 | 2 | 117 | 7 | 1 | 50 | 7 |
| Bites&Stings: Bednets for mosquitos | 30/08/2019 | 292 | 3 | 97 | 15 | 3 | 100 | 5 |
| Bites&Stings: Coils | 27/09/2019 | 353 | 1 | 353 | 10 | 1 | 100 | 10 |
| Bites&Stings: Environmental cleanliness & hygiene for scabies | 28/10/2019 | 1048 | 2 | 524 | 23 | 2 | 100 | 12 |
| Bites&Stings: FA for snakebites | 4/10/2019 | 1690 | 1 | 1690 | 18 | 1 | 100 | 18 |
| Bites&Stings: Hot water for sting of marine animals | 18/07/2019 | 601 | 1 | 601 | 0 | 0 | 0 | N/A |
| Limbs: Muscle cramps massage | 10/03/2020 | 1465 | 2 | 733 | 48 | 2 | 100 | 24 |
| Limbs: Stretching and warmup | 17/02/2020 | 2899 | 1 | 2899 | 197 | 1 | 100 | 197 |
| Limbs: Bracing and taping | 13/02/2020 | 416 | 3 | 139 | 38 | 3 | 100 | 13 |
| Limbs: Ice Elevation | 7/02/2020 | 1748 | 6 | 291 | 48 | 4 | 67 | 12 |
| Stomach and back: Dysmenorrhea | 25/05/2020 | 1251 | 2 | 626 | 99 | 2 | 100 | 50 |
| Stomach and back: Lift technique | 26/05/2020 | 1431 | 5 | 286 | 64 | 3 | 60 | 21 |
| Stomach and back: Walking for back pain | 16/06/2020 | 1120 | 1 | 1120 | 57 | 1 | 100 | 57 |
| Stomach and back: Heat for back pain | 13/06/2020 | 2253 | 1 | 2253 | 111 | 1 | 100 | 111 |
| Poisoning: Safe storage of toxics | 2/04/2020 | 660 | 2 | 330 | 25 | 2 | 100 | 13 |
| Heat and cold: Frostbite clothing | 4/06/2020 | 128 | 1 | 128 | 2 | 1 | 100 | 2 |
| Heat and cold: RF heatstroke | 15/05/2020 | 487 | 1 | 487 | 8 | 1 | 100 | 8 |
| Travel illnesses: Compression stockings | 22/08/2019 | 948 | 1 | 948 | 45 | 1 | 100 | 45 |
| Drowning prevention | 5/05/2020 | 412 | 2 | 206 | 22 | 2 | 100 | 11 |
| Telephone antenatal care | 17/08/2020 | 876 | 3 | 292 | 30 | 3 | 100 | 10 |
| Maternal sleeping position during pregnancy | 3/08/2020 | 475 | 1 | 475 | 34 | 1 | 100 | 34 |
| Safe delivery kit | 6/08/2020 | 125 | 2 | 63 | 9 | 2 | 100 | 5 |
| Uterine massage | 6/08/2020 | 277 | 2 | 139 | 36 | 2 | 100 | 18 |
| Prevention of bullet wounds | 19/08/2020 | 831 | 2 | 416 | 24 | 2 | 100 | 12 |
| Burns: Aloe vera for burns | 31/08/2020 | 284 | 4 | 142 | 21 | 4 | 100 | 5 |
| Palpation for detection of fever | 7/9/2020 | 1258 | 2 | 629 | 23 | 2 | 100 | 21 |
| Polymer-based ORS for diarrhoea | 15/9/2020 | 299 | 1 | 299 | 12 | 1 | 100 | 15 |

### Intervention study filter PubMed

| **Evidence summary [1]** | **Original search date** | **Number of records retrieved from PubMed without search filter** | **Relevant intervention studies identified without search filter** | **Number needed to read without search filter** | **Number of records retrieved from PubMed when using filter** | **Relevant intervention studies identified when using filter** | **Recall (%)** | **Number needed to read when using filter** |
| --- | --- | --- | --- | --- | --- | --- | --- | --- |
| Disinfection & wound irrigation of skin wounds | 10/01/2019 | 258 | 5 | 52 | 110 | 5 | 100 | 22 |
| Helmet removal | 14/01/2019 | 326 | 4 | 82 | 110 | 2 | 50 | 55 |
| Chin lift vs jaw thrust | 21/01/2019 | 64 | 1 | 64 | 32 | 0 | 0 | N/A |
| Posture for dyspnoea | 16/01/2019 | 1104 | 3 | 368 | 333 | 1 | 33 | 333 |
| Recovery position in spine injury | 21/01/2019 | 33 | 3 | 11 | 12 | 1 | 33 | 12 |
| Manual blood pressure monitoring | 21/01/2019 | 1017 | 5 | 203 | 636 | 4 | 80 | 159 |
| Transport spinal injury | 20/11/2018 | 1812 | 34 | 53 | 572 | 28 | 82 | 20 |
| Burns: deroofing or aspiration of blisters | 20/11/2019 | 464 | 1 | 464 | 113 | 0 | 0 | N/A |
| Burns: Eye decontamination after pepper spray | 21/11/2019 | 97 | 2 | 49 | 23 | 2 | 100 | 12 |
| Burns: Hydration for sunburn | 20/11/2019 | 137 | 2 | 69 | 46 | 2 | 100 | 23 |
| Burns: Sunscreen(+insect repellent) for sunburn | 15/11/2019 | 1516 | 10 | 152 | 485 | 9 | 90 | 54 |
| Burns: Polypodium leucotomos | 19/11/2019 | 16 | 4 | 4 | 7 | 4 | 100 | 2 |
| Bites&Stings: Household treatments for bee/wasp sting | 6/06/2019 | 201 | 1 | 201 | 32 | 1 | 100 | 32 |
| Bites&Stings: Stinger removal for bee/wasp sting | 22/07/2019 | 403 | 1 | 403 | 90 | 1 | 100 | 90 |
| Bites&Stings: Topical aspirin for bee/wasp sting | 10/07/2019 | 12 | 1 | 12 | 3 | 1 | 100 | 3 |
| Bites&Stings: Dog bite prevention | 27/09/2019 | 1131 | 6 | 189 | 230 | 6 | 100 | 38 |
| Bites&Stings: Wristbands for insect stings | 24/10/2019 | 11 | 3 | 4 | 9 | 3 | 100 | 3 |
| Bites&Stings: Washing/wet comb for lice | 17/10/2019 | 264 | 10 | 26 | 99 | 8 | 80 | 12 |
| Bites&Stings: Pediculicides | 17/10/2019 | 818 | 15 | 55 | 249 | 15 | 100 | 17 |
| Bites&Stings: Candles | 18/09/2019 | 35 | 4 | 9 | 6 | 4 | 100 | 2 |
| Bites&Stings: Ice for scorpion sting | 8/10/2019 | 114 | 1 | 114 | 21 | 1 | 100 | 21 |

### Intervention study filter Embase

| **Evidence summary [1]** | **Original search date** | **Number of records retrieved from Embase without search filter** | **Relevant intervention studies identified without search filter** | **Number needed to read without search filter** | **Number of records retrieved from Embase when using filter** | **Relevant intervention studies identified when using filter** | **Recall (%)** | **Number needed to read when using filter** |
| --- | --- | --- | --- | --- | --- | --- | --- | --- |
| Disinfection & wound irrigation of skin wounds | 10/01/2019 | 122 | 4 | 31 | 65 | 3 | 75 | 19 |
| Helmet removal | 14/01/2019 | 399 | 4 | 100 | 246 | 2 | 50 | 77 |
| Chin lift vs jaw thrust | 21/01/2019 | 67 | 1 | 67 | 25 | 1 | 100 | 42 |
| Posture for dyspnoea | 16/01/2019 | 2792 | 2 | 1396 | 1691 | 2 | 100 | 551 |
| Recovery position in spine injury | 21/01/2019 | 69 | 3 | 23 | 41 | 1 | 33 | 28 |
| Manual blood pressure monitoring | 21/01/2019 | 1687 | 4 | 422 | 563 | 4 | 100 | 281 |
| Transport spinal injury | 20/11/2018 | 1717 | 31 | 55 | 1156 | 24 | 77 | 23 |
| Burns: deroofing or aspiration of blisters | 20/11/2019 | 1223 | 1 | 1223 | 873 | 0 | 0 | N/A |
| Burns: Eye decontamination after pepper spray | 21/11/2019 | 159 | 2 | 80 | 95 | 2 | 100 | 32 |
| Burns: Hydration for sunburn | 20/11/2019 | 213 | 3 | 71 | 137 | 3 | 100 | 25 |
| Burns: Sunscreen(+insect repellent) for sunburn | 15/11/2019 | 1880 | 6 | 313 | 1287 | 6 | 100 | 99 |
| Burns: Polypodium leucotomos | 19/11/2019 | 30 | 3 | 10 | 18 | 2 | 67 | 6 |
| Bites&Stings: Household treatments for bee/wasp sting | 6/06/2019 | 311 | 1 | 311 | 239 | 1 | 100 | 72 |
| Bites&Stings: Stinger removal for bee/wasp sting | 22/07/2019 | 572 | 1 | 572 | 432 | 0 | 0 | N/A |
| Bites&Stings: Topical aspirin for bee/wasp sting | 10/07/2019 | 59 | 1 | 59 | 50 | 1 | 100 | 9 |
| Bites&Stings: Dog bite prevention | 27/09/2019 | 1482 | 5 | 296 | 1016 | 5 | 100 | 93 |
| Bites&Stings: Wristbands for insect stings | 24/10/2019 | 21 | 3 | 7 | 10 | 3 | 100 | 4 |
| Bites&Stings: Washing/wet comb for lice | 17/10/2019 | 234 | 10 | 23 | 115 | 8 | 80 | 15 |
| Bites&Stings: Pediculicides | 17/10/2019 | 1336 | 13 | 103 | 937 | 12 | 92 | 33 |
| Bites&Stings: Candles | 18/09/2019 | 44 | 4 | 11 | 29 | 4 | 100 | 4 |
| Bites&Stings: Ice for scorpion sting | 8/10/2019 | 170 | 1 | 170 | 125 | 1 | 100 | 45 |

### Observational study filter PubMed

| **Evidence summary [1]** | **Original search date** | **Number of records retrieved from PubMed without search filter** | **Relevant observational studies identified without search filter** | **Number needed to read without search filter** | **Number of records retrieved from PubMed when using filter** | **Relevant observational studies identified when using filter** | **Recall (%)** | **Number needed to read when using filter** |
| --- | --- | --- | --- | --- | --- | --- | --- | --- |
| Psychological First Aid | 10/12/2018 | 129 | 1 | 129 | 30 | 1 | 100 | 30 |
| ABCDE | 26/11/2018 | 88 | 1 | 88 | 28 | 1 | 100 | 28 |
| Crush injury | 4/01/2019 | 619 | 2 | 310 | 91 | 2 | 100 | 46 |
| Manual blood pressure monitoring | 21/01/2019 | 1017 | 1 | 1017 | 300 | 1 | 100 | 300 |
| Burns: timing of cooling | 23/12/2019 | 769 | 4 | 192 | 191 | 3 | 75 | 64 |
| Burns: RF for complications | 6/01/2020 | 674 | 7 | 96 | 455 | 4 | 57 | 114 |
| Burns: Chemical burn: irrigation | 22/11/2019 | 725 | 5 | 145 | 121 | 2 | 40 | 61 |
| Bites&Stings: RF dog bite | 1/10/2019 | 444 | 3 | 148 | 199 | 3 | 100 | 66 |
| Bites&Stings: RF infection dog/cat bites/scratch | 16/09/2019 | 806 | 6 | 134 | 358 | 6 | 100 | 60 |
| Bites&Stings: Irrigation dog/cat bites/scratch | 18/09/2019 | 325 | 1 | 325 | 112 | 1 | 100 | 112 |
| Bites&Stings: Stagant water RF | 2/10/2019 | 492 | 1 | 492 | 207 | 1 | 100 | 207 |
| Bites&Stings: Environmental cleanliness & hygiene for scabies | 28/10/2019 | 683 | 6 | 114 | 161 | 5 | 83 | 32 |
| Burns: Eye protection | 26/11/2019 | 590 | 2 | 295 | 98 | 1 | 50 | 98 |
| SIDS risk factors | 7/01/2020 | 1021 | 21 | 49 | 375 | 21 | 100 | 18 |
| SIDS home monitoring device | 7/07/2020 | 175 | 1 | 175 | 47 | 1 | 100 | 47 |
| Headrest for wiplash | 20/02/2020 | 116 | 2 | 58 | 18 | 1 | 50 | 18 |
| Object in mouth for epilepsy | 21/01/2020 | 168 | 2 | 84 | 73 | 2 | 100 | 37 |
| Risk factors for epilepsy | 23/01/2020 | 737 | 7 | 105 | 191 | 5 | 71 | 38 |
| Dysmenorrhea | 25/05/2020 | 550 | 7 | 79 | 237 | 6 | 86 | 40 |
| Breastfeeding for dehydration | 26/05/2020 | 626 | 4 | 157 | 258 | 4 | 100 | 65 |

### Observational study filter Embase

| **Evidence summary [1]** | **Original search date** | **Number of records retrieved from Embase without search filter** | **Relevant observational studies identified without search filter** | **Number needed to read without search filter** | **Number of records retrieved from Embase when using filter** | **Relevant observational studies identified when using filter** | **Recall (%)** | **Number needed to read when using filter** |
| --- | --- | --- | --- | --- | --- | --- | --- | --- |
| ABCDE | 26/11/2018 | 212 | 1 | 212 | 111 | 1 | 100 | 111 |
| Crush injury | 4/01/2019 | 302 | 2 | 151 | 93 | 2 | 100 | 47 |
| Posture for myocardial infarction | 22/01/2019 | 1598 | 1 | 1598 | 949 | 1 | 100 | 949 |
| Manual blood pressure monitoring | 21/01/2019 | 1687 | 1 | 1687 | 1057 | 1 | 100 | 1057 |
| Burns: RF for complications | 6/01/2020 | 790 | 5 | 158 | 576 | 4 | 80 | 144 |
| Burns: Cooling with ice | 18/12/2019 | 691 | 1 | 691 | 290 | 1 | 100 | 290 |
| Burns: Chemical burn: irrigation | 22/11/2019 | 388 | 3 | 129 | 207 | 3 | 100 | 69 |
| Bites&Stings: RF dog bite | 1/10/2019 | 426 | 3 | 142 | 238 | 3 | 100 | 79 |
| Bites&Stings: RF infection dog/cat bites/scratch | 16/09/2019 | 1190 | 6 | 198 | 633 | 6 | 100 | 106 |
| Bites&Stings: Irrigation dog/cat bites/scratch | 18/09/2019 | 379 | 1 | 379 | 218 | 1 | 100 | 218 |
| Bites&Stings: Stagant water RF | 2/10/2019 | 769 | 1 | 769 | 363 | 1 | 100 | 363 |
| Bites&Stings: Environmental cleanliness & hygiene for scabies | 28/10/2019 | 1048 | 4 | 262 | 438 | 3 | 75 | 146 |
| Bites&Stings: Sting removal of marine animals | 9/07/2019 | 109 | 1 | 109 | 33 | 1 | 100 | 33 |
| Skin wounds: RF for tetanus | 4/12/2019 | 912 | 1 | 912 | 470 | 1 | 100 | 470 |
| Burns: Eye protection | 26/11/2019 | 1145 | 3 | 382 | 337 | 3 | 100 | 112 |
| SIDS risk factors | 7/01/2020 | 1073 | 20 | 54 | 490 | 20 | 100 | 25 |
| SIDS home monitoring device | 7/07/2020 | 298 | 1 | 298 | 120 | 1 | 100 | 120 |
| Headrest for wiplash | 20/02/2020 | 125 | 2 | 63 | 31 | 1 | 50 | 31 |
| Object in mouth for epilepsy | 21/01/2020 | 505 | 2 | 253 | 348 | 2 | 100 | 174 |
| Risk factors for epilepsy | 23/01/2020 | 1412 | 7 | 202 | 833 | 7 | 100 | 119 |
| Dysmenorrhea | 25/05/2020 | 1251 | 7 | 179 | 764 | 7 | 100 | 109 |
| Breastfeeding for dehydration | 26/05/2020 | 786 | 4 | 197 | 479 | 4 | 100 | 120 |

1. Centre for Evidence-Based Practice (CEBaP). First Aid Evidence Summaries [Internet]. Mechelen, Belgium: Belgian Red Cross [cited Nov 19th 2020]. <<https://www.cebap.org/knowledge-dissemination/first-aid-evidence-summaries/>>.
