# Supplementary material for: Translation and validation of PubMed and Embase search filters for identification of systematic reviews, intervention studies, and observational studies in the field of first aid
Source: J Med Libr Assoc. 2021 Oct 1;109(4):599–608. doi: 10.5195/jmla.2021.1219 (PMC8608173; doi:10.5195/jmla.2021.1219)
Supplement: Supplementary file 3 — Appendix 3: Optimization of existing intervention study search filter for PubMed & Embase [file jmla-109-4-599-s03.docx]

## Appendix 3: Optimization of existing intervention study search filter for PubMed & Embase

| **Evidence summary [1]** | **Study** | **Study design** | **Retrieved from PubMed?** | **Potential search terms identified for addition to PubMed filter** | **Retrieved from Embase?** | **Potential search terms identified for addition to Embase filter** |
| --- | --- | --- | --- | --- | --- | --- |
| Helmet removal | Gastel 1998 | Non-RCT, within subjects | No | repeat*[TIAB] | No | repeat*:ab,ti |
| Helmet removal | Palumbo 1996 | Non-RCT, within subjects | No | compar*[TIAB] | No | ‘human experiment’/exp  compar*:ab,ti |
| Recovery position in spine injury | Blake 2002 | Non-RCT, within subjects | Yes |  | No | compar*:ab,ti |
| Chin lift vs jaw thrust | Prasarn 2014 | Non-RCT, within subjects | No | trials[TIAB] | Yes |  |
| Posture for dyspnoea | Perino 2016 | RCT, within subjects | No | *None identified: no methodology-related indexing, nor relevant free text words available* | *Not retrieved with gold standard search in Embase* |  |
| Posture for dyspnoea | Vitacca 2016 | RCT, within subjects | No | *None identified: Indexed as “Prospective studies”[Mesh], no relevant free text words available* | Yes |  |
| Recovery position in spinal injury | Del Rossi 2014 | RCT, within subjects | No | compar*[TIAB] | No | compar*:ab,ti |
| Recovery position in spinal injury | Hyldmo 2014 | RCT, within subjects | No | compar*[TIAB] | Yes |  |
| Manual blood pressure monitoring | Myers 2009 | Non-RCT, within subjects | No | compar*[TIAB] | Yes |  |
| Spinal immobilization | Del Rossi 2004 | Non-RCT, within subjects | No | repeat*[TIAB] | No | repeat*:ab,ti |
| Spinal immobilization | DuBose 2016 | Non-RCT, within subjects | No | *None identified: no methodology-related indexing, nor relevant free text words available* | No | *None identified: no methodology-related indexing, nor relevant free text words available* |
| Spinal immobilization | Edlich 2011 | Non-RCT, within subjects | No | *None identified: no methodology-related indexing, nor relevant free text words available* | No | *None identified: no methodology-related indexing, nor relevant free text words available* |
| Spinal immobilization | Nemunaitis 2015 | RCT, within subjects | No | “Evaluation study”[PT] | No | ‘Evaluation study’/exp |
| Spinal immobilization | Prasarn 2012a | Non-RCT, within subjects | Yes |  | No | ‘human experiment’/exp  compar*:ab,ti |
| Spinal immobilization | Prasarn 2012b | Non-RCT, within subjects | No | versus | Yes |  |
| Spinal immobilization | Prasarn 2017 | RCT, within subjects | Yes |  | No | compar*:ab,ti |
| Deroofing burn blisters | Swain 1987 | Non-RCT | No | *None identified: no methodology-related indexing, nor relevant free text words available* | No | *None identified: no methodology-related indexing, nor relevant free text words available* |
| Burns Polypodium Leucotomos | Middelkamp-Hup 2004 | Non-RCT, within subjects | Yes |  | No | ‘controlled study’/de |
| Bee sting removal | Visscher 1996 | RCT, within subjects | Yes |  | No | ‘controlled study’/de |
| Sunscreen (+ insect repellent) | Montemarano 1997 | RCT | No | *None identified: Indexed as “Letter”[PT], no abstract available* | *Not retrieved with gold standard search in Embase* |  |
| Washing & wet combing for lice | Izri 2016 | Non-RCT | No | “Evaluation study”[PT]  compar*[TIAB] | No | ‘evaluation study’/exp  compar*:ab,ti |
| Washing & wet combing for lice | Speare 2003 | Non-RCT | No | *None identified: no methodology-related indexing, nor relevant free text words available* | No | *None identified: indexed as ‘conference paper’/exp, no relevant free text words available* |
| Pediculicides | Taplin 1982 | RCT | Yes |  | No | ‘controlled study’/exp  ‘double blind’:ab,ti  compar*:ab,ti |

1. Centre for Evidence-Based Practice (CEBaP). First Aid Evidence Summaries [Internet]. Mechelen, Belgium: Belgian Red Cross [cited Nov 19th 2020]. <<https://www.cebap.org/knowledge-dissemination/first-aid-evidence-summaries/>>.
