# Supplementary material for: Translation and validation of PubMed and Embase search filters for identification of systematic reviews, intervention studies, and observational studies in the field of first aid
Source: J Med Libr Assoc. 2021 Oct 1;109(4):599–608. doi: 10.5195/jmla.2021.1219 (PMC8608173; doi:10.5195/jmla.2021.1219)
Supplement: Supplementary file 4 — Appendix 4: Validation reference gold standards composition [file jmla-109-4-599-s04.docx]

## Appendix 4: Validation reference gold standards composition

### Intervention study filter PubMed

| **Evidence summary [1]** | **Original search date** | **Number of records retrieved from PubMed without search filter** | **Relevant intervention studies identified without search filter** | **Number needed to read without search filter** | **Number of records retrieved from PubMed when using original filter** | **Relevant intervention studies identified when using original filter** | **Recall original filter (%)** | **Number needed to read when using original filter** | **Number of records retrieved from PubMed when using adapted filter** | **Relevant intervention studies identified when using adapted filter** | **Recall adapted filter (%)** | **Number needed to read when using adapted filter** |
| --- | --- | --- | --- | --- | --- | --- | --- | --- | --- | --- | --- | --- |
| Bites&Stings: Impregnated clothing | 1/08/2019 | 423 | 1 | 423 | 112 | 0 | 0 | N/A | 142 | 0 | 0 | N/A |
| Limbs: Muscle cramps massage | 10/03/2020 | 999 | 4 | 250 | 323 | 4 | 100 | 81 | 424 | 4 | 100 | 106 |
| Limbs: Stretching and warmup | 17/02/2020 | 1273 | 10 | 127 | 563 | 10 | 100 | 56 | 696 | 10 | 100 | 70 |
| Limbs: Bracing and taping | 13/02/2020 | 267 | 1 | 267 | 146 | 1 | 100 | 146 | 175 | 1 | 100 | 175 |
| Limbs: Ice Elevation | 7/02/2020 | 1488 | 7 | 213 | 516 | 7 | 100 | 74 | 710 | 7 | 100 | 101 |
| Stomach and back: Dysmenorrhea | 25/05/2020 | 550 | 7 | 79 | 267 | 7 | 100 | 38 | 319 | 7 | 100 | 46 |
| Stomach and back: Heat for back pain | 13/06/2020 | 1006 | 7 | 144 | 467 | 7 | 100 | 67 | 599 | 7 | 100 | 86 |
| Poisoning: Safe storage of toxics | 2/04/2020 | 590 | 1 | 590 | 156 | 1 | 100 | 156 | 207 | 1 | 100 | 207 |
| Telephone antenatal care | 17/08/2020 | 463 | 5 | 93 | 277 | 5 | 100 | 55 | 332 | 5 | 100 | 66 |
| Prevention of bullet wounds | 19/08/2020 | 767 | 3 | 256 | 225 | 2 | 67 | 113 | 304 | 2 | 67 | 152 |
| Burns: Aloe vera for burns | 31/08/2020 | 103 | 3 | 34 | 41 | 2 | 67 | 21 | 49 | 3 | 100 | 16 |
| Jellyfish stings FA | 11/09/2020 | 476 | 5 | 95 | 35 | 5 | 100 | 7 | 56 | 5 | 100 | 11 |
| Zinc ORS diarrhoea | 11/09/2020 | 353 | 3 | 118 | 156 | 3 | 100 | 52 | 204 | 3 | 100 | 68 |
| Skin wound covers | 21/11/2019 | 1240 | 2 | 620 | 330 | 0 | 0 | N/A | 415 | 0 | 0 | N/A |
| Socks for friction blisters | 18/12/2019 | 25 | 4 | 6 | 5 | 4 | 100 | 1 | 12 | 4 | 100 | 3 |
| Mouth covers for cough | 12/12/2019 | 379 | 4 | 95 | 238 | 2 | 50 | 119 | 259 | 2 | 50 | 130 |
| Honey for cough | 13/01/2020 | 81 | 5 | 16 | 36 | 5 | 100 | 7 | 40 | 5 | 100 | 8 |
| Hayfever irrigation | 15/01/2020 | 102 | 1 | 102 | 64 | 1 | 100 | 64 | 69 | 1 | 100 | 69 |

### Intervention study filter Embase

| **Evidence summary [1]** | **Original search date** | **Number of records retrieved from Embase without search filter** | **Relevant intervention studies identified without search filter** | **Number needed to read without search filter** | **Number of records retrieved from Embase when using original filter** | **Relevant intervention studies identified when using original filter** | **Recall original filter (%)** | **Number needed to read when using original filter** | **Number of records retrieved from Embase when using adapted filter** | **Relevant intervention studies identified when using adapted filter** | **Recall adapted filter (%)** | **Number needed to read when using adapted filter** |
| --- | --- | --- | --- | --- | --- | --- | --- | --- | --- | --- | --- | --- |
| Bites&Stings: Impregnated clothing | 1/08/2019 | 564 | 1 | 564 | 218 | 0 | 0 | N/A | 246 | 0 | 0 | N/A |
| Limbs: Muscle cramps massage | 10/03/2020 | 1465 | 3 | 488 | 624 | 3 | 100 | 208 | 784 | 3 | 100 | 261 |
| Limbs: Stretching and warmup | 17/02/2020 | 2899 | 9 | 322 | 1440 | 9 | 100 | 160 | 1716 | 9 | 100 | 191 |
| Limbs: Ice Elevation | 7/02/2020 | 1748 | 5 | 350 | 674 | 3 | 60 | 225 | 916 | 5 | 100 | 183 |
| Stomach and back: Dysmenorrhea | 25/05/2020 | 1251 | 9 | 139 | 671 | 8 | 89 | 84 | 804 | 9 | 100 | 89 |
| Stomach and back: Heat for back pain | 13/06/2020 | 2253 | 7 | 322 | 1181 | 7 | 100 | 169 | 1446 | 7 | 100 | 207 |
| Poisoning: Safe storage of toxics | 2/04/2020 | 660 | 1 | 660 | 335 | 1 | 100 | 335 | 402 | 1 | 100 | 402 |
| Telephone antenatal care | 17/08/2020 | 876 | 6 | 146 | 476 | 6 | 100 | 79 | 622 | 6 | 100 | 104 |
| Safe delivery kit | 6/08/2020 | 125 | 1 | 125 | 57 | 1 | 100 | 57 | 78 | 1 | 100 | 78 |
| Prevention of bullet wounds | 19/08/2020 | 831 | 1 | 831 | 307 | 1 | 100 | 307 | 430 | 1 | 100 | 430 |
| Burns: Aloe vera for burns | 31/08/2020 | 284 | 4 | 71 | 93 | 3 | 75 | 31 | 121 | 4 | 100 | 30 |
| Jellyfish stings FA | 11/09/2020 | 422 | 5 | 84 | 47 | 5 | 100 | 9 | 69 | 5 | 100 | 14 |
| Zinc ORS diarrhoea | 11/09/2020 | 528 | 3 | 176 | 276 | 3 | 100 | 92 | 340 | 3 | 100 | 113 |
| Skin wound covers | 21/11/2019 | 1852 | 2 | 926 | 556 | 1 | 50 | 556 | 711 | 1 | 50 | 711 |
| Socks for friction blisters | 18/12/2019 | 27 | 4 | 129 | 7 | 3 | 75 | 2 | 15 | 4 | 100 | 4 |
| Mouth covers for cough | 12/12/2019 | 515 | 3 | 76 | 270 | 3 | 100 | 90 | 324 | 3 | 100 | 108 |
| Honey for cough | 13/01/2020 | 228 | 5 | 46 | 86 | 5 | 100 | 17 | 100 | 5 | 100 | 20 |
| Hayfever irrigation | 15/01/2020 | 128 | 1 | 128 | 85 | 1 | 100 | 85 | 93 | 1 | 100 | 93 |

1. Centre for Evidence-Based Practice (CEBaP). First Aid Evidence Summaries [Internet]. Mechelen, Belgium: Belgian Red Cross [cited Nov 19th 2020]. <<https://www.cebap.org/knowledge-dissemination/first-aid-evidence-summaries/>>.
